# A Rare Onset of T-Lymphoid Blast Crisis in Chronic Myeloid Leukemia with Two Distinct Blast Populations

**DOI:** 10.3390/hematolrep16030040

**Published:** 2024-06-27

**Authors:** Alessandra Mongia, Francesca Romano, Sara Ciullini Mannurita, Benedetta Peruzzi, Sara Bencini, Daniela Parrini, Laura Fasano, Alessandra Fanelli

**Affiliations:** 1 General Laboratory, Careggi University Hospital, Largo Brambilla 3, 50134 Florence, Italy; mongiaa@aou-careggi.toscana.it (A.M.); ciullinimannuritas@aou-careggi.toscana.it (S.C.M.); fanellia@aou-careggi.toscana.it (A.F.); 2 Flow Cytometric and Immunotherapeutic Diagnostic Center, Careggi University Hospital, Largo Brambilla 3, 50134 Florence, Italy; peruzzib@aou-careggi.toscana.it (B.P.); bencinis@aou-careggi.toscana.it (S.B.); 3 Genetic Diagnosis Service, Careggi University Hospital, Largo Brambilla 3, 50134 Florence, Italy; parrinid@aou-careggi.toscana.it; 4 Hematology Unit, Careggi University Hospital, Largo Brambilla 3, 50134 Florence, Italy; laura.fasano@unifi.it

**Keywords:** chronic myeloid leukemia, T-lymphoid blastic crisis

## Abstract

Chronic myeloid leukemia (CML) is a myeloproliferative neoplasm characterized by bone marrow expansion and the proliferation of one or more myeloid cell lineages, predominantly driven by the expression of the constitutively active fusion product tyrosine kinase BCR:ABL1. Rarely, CML patients directly develop a blast crisis (BC), mostly of myeloid origin. CML at blast crisis with a T-cell phenotype at diagnosis, without any prior history of CML, is extremely rare. Herein, we describe one rare CML case, in a young man showing an unusual and early T-lymphoid blastic crisis at diagnosis, as the first onset of a previously unknown CML. The multidisciplinary collaboration between laboratorians and clinicians for the diagnosis and management of this atypical case was crucial in outlining both a targeted pharmacological treatment and a successful hematopoietic stem cell transplantation.

## 1. Introduction

Chronic myeloid leukemia (CML) is a clonal malignant myeloproliferative expansion of hematopoietic progenitor cells, and it can involve myeloid, monocytic, erythroid, megakaryocytic, B-lymphoid, and, occasionally, T-lymphoid lineages [1].

In CML, the presence of the Philadelphia (Ph) chromosome, resulting from the t(9; 22) translocation and harboring the BCR-ABL1 fusion gene, is directly linked to leukemogenesis [1]. This gene encodes for a tyrosine phosphokinase protein that stimulates predominantly granulocyte production and leads to genomic instability.

CML occurs in about 0.7 to 1 in 100,000 individuals per year according to several European registries and involves mainly patients in their 40s to 70s, with a male predominance [2].

CML is a triphasic disorder. The majority of patients present in chronic phase (CP), followed by an accelerated phase (AP) and a blast phase (BP), if untreated. The latter can appear either as myeloid (in 70–80% of cases) or as lymphoid blast crisis (BC), with B-cell lineage being more common [3,4]. T-cell lymphoid blast crisis in CML represents a rare subset, characterized by the expansion of immature T lymphocytes resembling lymphoblasts, indicating an advanced stage with poor prognosis and limited treatment options.

The predisposing risk factors are unknown; however, it has been observed that there is an increased incidence of three major types of leukemia (acute lymphocytic leukemia, acute myeloid leukemia, and chronic myeloid leukemia) in relation to ionizing radiation exposure and based on age [5].

CML often presents with nonspecific symptoms, and the quality of life in patients has strongly improved thanks to tyrosine kinase inhibitors (TKIs) therapy: the 8-year overall survival is now 87% [6].

TKIs provide indeed excellent outcomes for patients with CP-CML, with most patients expected to achieve a normal life expectancy. However, responses to TKIs in patients with BP-CML are frequently short-lived, leading to high mortality rates attributed to disease refractoriness within one year of diagnosis [7].

In this case report, we discuss the case of a young male patient who presented at diagnosis an atypical phase of T-lymphoid blast crisis of CML without previously reported distinct features.

## 2. Case Report

A 40-year-old male patient, admitted to the Emergency Department for pallor, skin rash, and facial swelling, presented hepatosplenomegaly and bilateral multidistrict lymphadenopathies involving the submandibular, laterocervical, axillary, and inguinal regions; in addition, he presented profuse night sweats associated with fatigue; no weight loss was reported. The patient tested positive for severe acute respiratory syndrome coronavirus 2 (SARS-CoV-2) with mild symptoms and was hospitalized in the infectious diseases department.

Biochemical parameters, performed on a Roche Cobas^®^ 8000 (Roche, Basel, Switzerland), showed no critical abnormalities, except for LDH high levels (825 U/L).

Peripheral whole blood cell count was performed on a Sysmex XN-9100^TM^ automatic hematology analyzer (Sysmex Corporation, Kobe, Japan). Whole blood cell count showed leukocytosis (WBC 310 × 10^9^/L), anemia (Hb 8.2 g/dL), and thrombocytopenia (PLT 85 × 10^9^/L). The differential leukocyte count channel showed an anomalous WBC scattergram, with unseparated cell clusters and flags indicating the presence of atypical lymphocytes and the presence of blasts cells.

**Figure 1:**
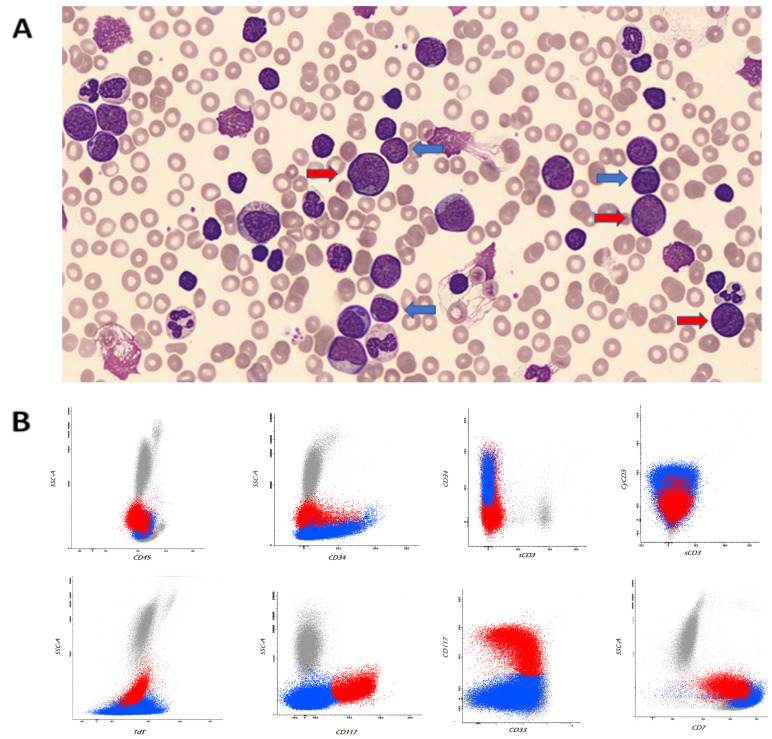
Peripheral blood leukocytosis with left shift and blasts of different sizes (blue arrows indicate immature T-lymphoid cells and red arrows indicate immature myeloid cells) (May–Grünwald Giemsa stain, ×100) (**A**). Representative flow cytometry dot plot graphs showing two abnormal populations: one characterized by immature T-lymphoid phenotype (CD34+, TdT+, cyCD3+, sCD3−) in blue and one with characteristics of immature myeloid phenotype (CD117+, CD33+) and CD7 aberrant expression in red (**B**).

A peripheral blood smear was performed using an automatic smear-making/staining device (Sysmex SP-1000i^TM^), and the microscopic revision of the blood smear showed a double pathological cell population with blast appearance showing immature chromatin, basophilic cytoplasm, and evident nucleoli (62% of total cells): a small one with lymphoid characteristics and a second one of larger size, sometimes with fine granulations (see Figure 1A).

A comprehensive flow-cytometry antigen panel was used for the immunophenotypic characterization.

The immunophenotypic markers included in the analysis of the bone marrow at diagnosis were as follows: CD1a, CD2, CD3 (surface vs cytoplasmic), CD4, CD5, CD7, CD8, CD13, CD14, CD15, CD16, CD19, CD33, CD34, CD56, CD64, CD65, CD117, CD300, HLA-DR, TCR alfa/beta, TCR gamma/delta, TdT, and myeloperoxidase (MPO). Data acquisition was carried out on a BD FACSCanto^TM^ II (BD Biosciences, San Jose, CA, USA) equipped with BD FACSDiVA^TM^ software (version 1.8).

The antigen profile confirmed the presence of two abnormal cell populations: a first one (50% of total cells) with immature T-lymphoid phenotype (CD34+, TdT+, cyCD3+, sCD3−), CD5+dim, and absent expression of CD1a and CD8, and a second one (20% of total cells) with immature myeloid progenitors’ phenotype (CD117+, CD33+) showing an aberrant CD7 expression (see Figure 1B and Figure S1 supplementary).

**Figure 2:**
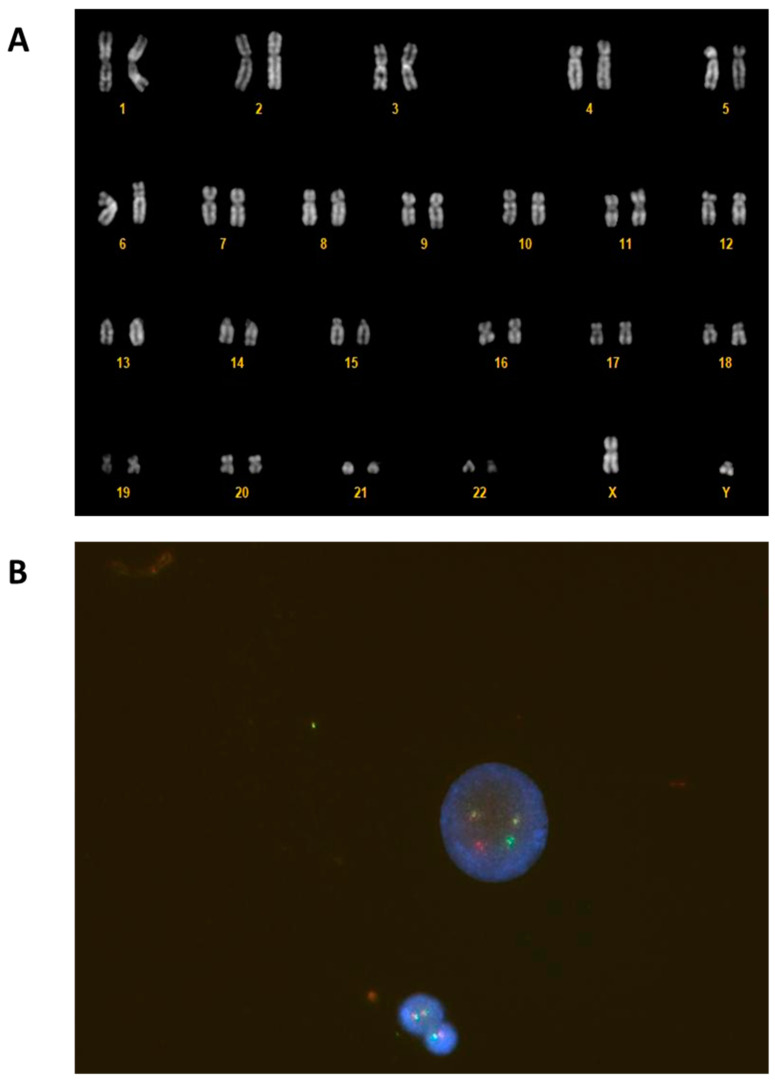
G-banded karyotype on cells from a BM sample with the classic Philadelphia translocation t(9; 22)(q34; q11) (**A**). Fluorescence in situ hybridization (FISH) showed BCR:ABL1 fusion on interphase nuclei with a dual fusion translocation probe (**B**).

The cytogenetic analysis was performed on a bone marrow (BM) sample 24 h after the culture was harvested, and the slides were GTG banded. Fluorescence in situ hybridization (FISH) was performed on an uncultured sample with MetaSystems^TM^ XL BCR/ABL probe (MetaSystems, Heidelberg, Germany). Both FISH and cytogenetic analysis showed the Philadelphia translocation (Figure 2).

The quantification of the BCR:ABL1 transcript was performed on BM by real-time quantitative polymerase chain reaction (RQ-PCR) using Applied Biosystems^TM^ Thermal Cycler (Applied Biosystems, Waltham, MA, USA) and Qiagen^®^ Ipsogen BCR-ABL1 Mbcr IS-MMR kit (Qiagen, Venlo, Netherlands).

Quantitative analysis of donor chimerism was performed on BM at different times, by next-generation sequencing (NGS) using Illumina MiSeq™ instrumentation (Illumina, San Diego, CA, USA).

Mutational analysis of BCR:ABL1 was performed by nested reverse transcriptase (RT) and subsequent direct sequencing on an Applied Biosystems^TM^ Genetic Analyzer.

After two weeks, the patient tested negative for SARS-CoV-2 and was transferred to the hematology department. During hospitalization, genetic analysis was performed.

Next-generation sequencing (NGS) analysis was done on the BM sample using an Ion GeneStudio^TM^ S5 System and Oncomine^TM^ Myeloid Assay kit (Thermo Fisher Scientific, Waltham, MA, USA).

NGS documented two somatic mutations in the WT1 gene: the previously described c.1372C>T; p.R458X [VAF: 43%] [8] and the unknown c.1108_1109insA; p.R370Qfs*15 [VAF: 42%].

Taken together, all these features were consistent with early T-lymphoid blast crisis of chronic myeloid leukemia.

**Figure 3:**
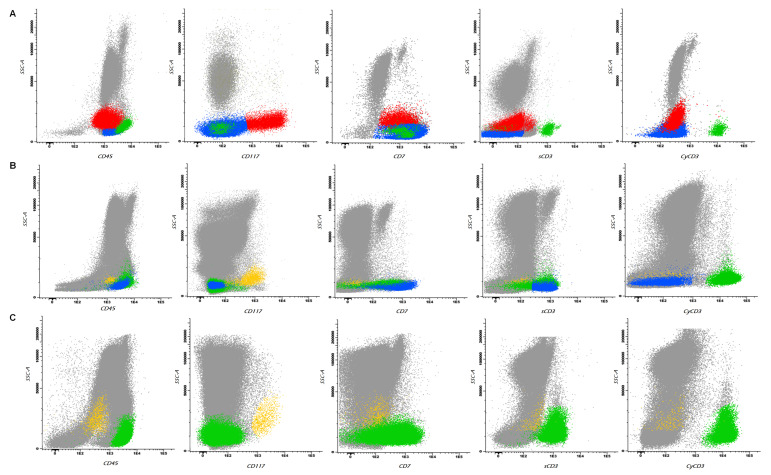
Multiparametric flow cytometry representations of BM analysis at different time points. Panel (**A**) shows at diagnosis two different abnormal populations: one characterized by immature T-lymphoid phenotype (CD34+, TdT+, cyCD3+, sCD3−) colored in blue and one with characteristics of immature myeloid phenotype (CD117+, CD33+) and CD7 aberrant expression colored in red. Mature T lymphocytes are colored in green. Panel (**B**) shows, after induction, persistent residual immature T-lymphoid cells colored in blue, whereas myeloid progenitors show no deviation from normal and are colored in orange. Mature T lymphocytes are colored in green. Panel (**C**) shows, after transplantation, negative measurable disease: immature T-lymphoid cells are no longer identified and myeloid progenitors, colored in orange, show no deviation from normal. Mature T lymphocytes are colored in green.

Flow cytometry analysis was also performed in BM samples in order to confirm the diagnosis and to better phenotypically characterize blast cells (see Figure 3A).

Because of the leukocytosis at onset, the patient was cytoreduced with prednisone and hydroxyurea; in order to avoid tumor lysis syndrome, he also started a prophylactic treatment with intravenous hydration and uricosuric agents. He was induced with a cyclophosphamide, idarubicin, vincristine, and dexamethasone regimen, based on the Italian GIMEMA LAL1913 protocol, supplemented with dasatinib [9].

The FISH study performed on the BM sample after the induction treatment revealed 63.0% of cells with fusion signals for the BCR:ABL1 gene, and flow cytometry analysis showed the presence of residual T-lymphoid phenotype blasts, while myeloid progenitors did not show aberrations (see Figure 3B).

The quantification of the BCR:ABL1 transcript was performed on BM at different time-points using RQ-PCR and detected the presence of subtype b2a2 p210 (see Table 1). BCR:ABL1 mutational analysis did not reveal any variants.

Considering these results, TKIs therapy was switched to ponatinib 45 mg per day.

In view of the suboptimal clearance of the BCR:ABL1 transcript, the patient underwent a second cycle of chemotherapy with methotrexate and high-dose cytarabine. Routine prophylactic intrathecal treatment, with cytarabine, methotrexate, and dexamethasone, was performed, demonstrating no central nervous system (CNS) involvement. After two cycles of chemotherapy, BCR:ABL1 transcript significantly decreased in bone marrow (ratio of BCR/ABL1 to ABL1 = 0.93%).

Five months after diagnosis, due to the underlying disease risk, allogeneic hematopoietic stem cell transplantation (HSCT) from a 10/10 matched unrelated donor (MUD) was performed using a myeloablative cyclophosphamide (120 mg/kg) and total body irradiation (1200 cGy) conditioning regimen. Maintenance treatment with ponatinib 30 mg per day was introduced sixty days after the transplant procedure. The dosage was subsequently reduced to 15 mg per day due to the development of neutropenia.

Follow-up bone marrow examinations, performed at different time-points, included morphological analysis, multiparametric flow cytometry (MFC), quantitative monitoring of BCR:ABL1 transcript, and the quantitative analysis of donor chimerism (see Table 1).

Multiparametric flow cytometry level of measurable residual disease (MRD) was determined using two different strategies: a “different from normal” approach for the myeloid compartment study and a MRD T-ALL approach to evaluate the immature T-lymphoid phenotype. T-lymphoblasts were not detectable by flow cytometry (LOD 0.0006%, LOQ 0.001%), and no “different from normal” cells were found by flow cytometry in the myeloid patterns of maturations demonstrating MFC-MRD negativity (Figure 3C). Moreover, follow-up BM examinations demonstrated barely or no detectable BCR/ABL1 fusion transcript levels, consistent with a deep molecular response (above MR 4.0). The same results were obtained on peripheral blood (PB).

Fifteen months after transplantation, the patient is in good health, leads a normal life, has no signs of GvHD (Graft versus Host Disease), and all hematologic parameters returned within normal ranges.

## 3. Discussion and Conclusions

Philadelphia-positive T-cell acute leukemias are extremely rare. According to the literature, approximately 50 cases have been described at present and include patients with de novo T-cell acute lymphoblastic leukemia (T-ALL) and/or T-lineage BP-CML [10,11,12,13,14]. Differential diagnosis between T-lineage BP-CML and de novo T-ALL can be very challenging, and our case is particularly interesting as it exhibited features of both: no prior history of CML and bone marrow involvement supported Ph+ T-ALL, whereas the patient’s age and extensive extramedullary involvement were consistent with T-cell BP-CML.

The early identification of two blast cell lines from a peripheral blood smear guided clinicians and laboratorians to the characterization of two different blast cell populations. Morphological examination, flow cytometry, and cytogenetics allowed for the rapid characterization of T-lymphoid blast crisis of CML. Particularly, the detection of the major p210 BCR breakpoint by cytogenetic analysis was crucial in establishing the final diagnosis.

The early diagnosis and the different characterization in bone marrow samples allowed for the selection of the most appropriate treatment and helped with performing an early HSCT in first remission. Although TKIs have transformed outcomes for CP-CML patients, there is currently no standard therapy for BP-CML, and prognosis remains poor for this subset [15]. Our patient underwent an unconventional therapeutic protocol tested in an Italian clinical trial, which, when combined with ponatinib, led to complete disease remission with a favorable safety profile. This result enabled us to proceed with the patient’s transplant under optimal conditions, thereby contributing to its long-term success.

This case confirmed the relevance of multidisciplinary collaboration for CML management. In fact, the patient had hematologic, cytogenetic, and molecular features present in both CML and ALL. All in-depth analyses described above were strongly indicative of a diagnosis of CML and were crucial in outlining a correct treatment plan for the patient. Additional cases may be useful to explore how to improve the diagnostic workflow for patients experiencing blast crisis of CML, facilitating a more precise characterization of the leukemic population and aiding in the selection of optimal therapeutic interventions to enhance patient outcomes.

## Figures and Tables

**Table 1:** Chimerism and minimal residual disease monitoring on bone marrow aspirate samples after allogeneic stem cell transplantation.

Months after Transplant	Complete Morphological Remission (CMR)	Multiparametric Flow Cytometry- Measurable Residual Disease (MFC-MRD)	BCR/ABL MR	Chimerism
1	Yes	Negative	MR4 (0.0037%)	99.7%
2	Yes	Negative	MR4.5 (0.0015%)	99.8%
3	Yes	Negative	MR4.5 (0.0016%)	99.9%
6	Yes	Negative	MR5	99.7%
12	Yes	Negative	Undetected	99.9%
15	Yes	Negative	Undetected	99.9%

## Data Availability

The authors declare that data supporting the findings of this study are available within the article.

## Supplementary Materials

The following supporting information can be downloaded at https://www.mdpi.com/article/10.3390/hematolrep16030040/s1: Figure S1: Multiparameter flow cytometry analysis of additional markers used for complete characterization.
