# Supplementary material for: A Rare Onset of T-Lymphoid Blast Crisis in Chronic Myeloid Leukemia with Two Distinct Blast Populations
Source: Hematol Rep. 2024 Jun 27;16(3):413–20. doi: 10.3390/hematolrep16030040 (PMC11431131; doi:10.3390/hematolrep16030040)
Supplement: Supplementary file 1 [file hematolrep-16-00040-s001.zip › hematolrep-2950310-supplementary.pdf]

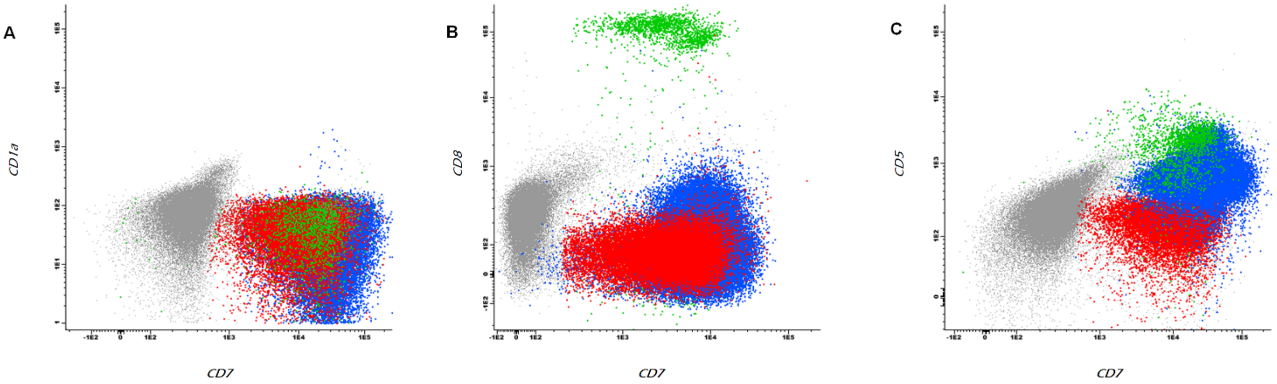

Supplementary Figure S1. Multiparameter flow cytometry analysis of additional markers used for complete characterization: immature T-lymphoid are colored in blue, blasts with characteristics of immature myeloid phenotype arboring CD7 aberrant expression are colored in red and mature T lymphocytes are colored in green.

Panel A shows negative expression for CD1a in blast populations and in T mature lymphocytes.

Panel B shows negative expression of CD8 in both blast populations, while a proportion of T mature lymphocytes shows expression of CD8.

Panel C shows a decreased CD5 expression in immature T-lymphoid blasts, negative CD5 expression on the immature myeloid phenotype, while CD5 is brilliant in the mature T lymphocytes.
